# On-Board Ship Detection for Medium Resolution Optical Sensors

**DOI:** 10.3390/s21093062

**Published:** 2021-04-28

**Authors:** Somnath Ghosh, Pramod Kumar Konugurthi, Gowri Shankar Rao Singupurapu, Shivi Patel, Tirupathi Tammanagari, Mallikarjuna Rao Desu, Lalit Krushna Thakar, Ishika Ghara

**Affiliations:** 1 Advanced Data Processing and Research Institute, Secunderabad 500009, India; somnath@adrin.res.in (S.G.); gowrishankar@adrin.res.in (G.S.R.S.); shivipatel@adrin.res.in (S.P.); tiru@adrin.res.in (T.T.); mallik@adrin.res.in (M.R.D.); 2 U.R. Rao Satellite Centre, Bengaluru 560017, India; ljthakar@ursc.gov.in (L.K.T.); ishika@ursc.gov.in (I.G.)

**Keywords:** remote sensing, ship detection, on-board processing, CFAR, satellite data processing, real-time processing, maritime surveillance, FPGA

## Abstract

In recent years there has been an increased interest in ocean surveillance. The activity includes control and monitoring of illegal fisheries, manmade ocean pollution and illegal sea traffic surveillance, etc. The key problem is how to identify ships and ship-like objects accurately and in a timely manner. In this context, currently, many solutions have been proposed based on high resolution optical and radar remote sensing systems. Most often, these systems suffer from two major limitations viz., limited swath, thereby requiring multiple satellites to cover the region of interest and huge volumes of data being transmitted to ground, even though effective per-pixel information content is minimal. Another limitation is that the existing systems are either simulated on ground or built using the non-space qualified/Commercial Of-The-Shelf (COTS) components. This paper proposes an efficient on-board ship detection system/package connected with medium resolution wide swath optical camera. The methodology adopted has three major components, viz., onboard data processing for improving the radiometric fidelity, followed by a ship detection using modified Constant False Alarm Rate algorithm (CFAR) and a false alarm suppression module to mask false identifications. Finally, the package outputs only the locations of the ships, which is transmitted to the ground. The proposed system reduces the effective volume of data to be transmitted and processed on ground and also significantly cuts down the turnaround time for achieving the end objective. The system is built on radiation hardened Field Programmable Gate Array (FPGA) devices to meet the various engineering constraints such as real-time performance, limited onboard power, radiation hardness, handling of multiple custom interfaces etc. The system is tested with one of the medium resolution Multispectral Visual and Near Infra-Red (MX-VNIR) sensor having a spatial resolution of around 50 m and swath of around 500 Kms, which would be flown with one of the upcoming satellites. The systems performance is also verified on ground with Indian Remote Sensing (IRS) Satellite’s Resourcesat’s Advanced Wide Field Sensor (AWiFS) data and the results are found to be quite encouraging as well as meeting the mission objectives.

## 1. Introduction

The monitoring of ships and ship-like objects or vessels in the sea plays an important role in maritime surveillance. Information related to vessel identification, its movement and intention is essential to several agencies including government bodies and organizations of the states. This enables various services including enhanced search-and-rescue operation, protection of off-shore resources, efficient management of Vessel Traffic Services (VTS) for safe and efficient navigation, deterrence of smuggling, drug trafficking and protection of the environment, etc. [1]. These various services demand monitoring of wide sea areas, harbor areas, docking areas, shipping lanes and offshore coastal areas. The frequency of observation and exact time to monitor is linked with the end application. Further, a few applications require the response time to be as low as possible. With the increase in the number of vessels, manual monitoring often becomes intractable, so a systematic observation of the sea surface and sub-surface areas of interest by all available and practical means is required for effective surveillance at sea.

A vast majority of maritime applications rely on the identification of the ship/vessels along with the time tagged location information provided in a timely manner. Currently agencies use a combination of ground-borne, sea-borne, air-borne and space-borne systems to provide this information. Ground-borne or seaborne systems are the simplest because of ease of installation and maintenance; however, they suffer from a limited coverage of the area of interest and a large number of stations along the coast and across the deep sea are essential to cover larger areas of interest. Airborne systems consist of a camera system installed on the belly of the aircraft and imaging the ground vertically down (near nadir). These systems provide near-nadir view with relatively wider coverage from higher altitude, but are expensive, difficult to maintain and involve huge amounts of post processing of the data on ground. Space-based systems have been considered more effective and optimal for systematic monitoring. In space-based systems, two methods are used viz., communication-based and imaging-based. In communication based systems Automatic Identification Systems (AIS) is the most common and quite popular system used worldwide, while in these imaging-based systems, a variety of sensors including optical and Synthetic Aperture Radar (SAR) systems are used with mission-specific orbits and platforms.

AIS [2,3,4] transceivers are installed on all the vessels to establish a dynamic network within their proximity. AIS-transceivers broadcast information about themselves at a regular time interval. The information includes the vessel’s unique identification number, position information, velocity, etc. However, the system poses a few limitations including limited range of operation, noisy communication channels (subject to climatic conditions), incorrect/wrong reporting and most importantly discretionary information sharing to the main network. The limitations of AIS can be overcome by deploying remote sensors with an appropriate platform and monitoring the region-of-interest periodically or on requirement basis.

Remote sensors in combination with AIS and ground-borne radar networks are widely adopted for a complete maritime surveillance. Conventionally, under satellite- based monitoring a ground-based setup is envisaged for data acquisition, processing, information extraction and dissemination. Often, sensors are placed in orbits ranging from ~300–36,000 km with a mission-specific inclination angle. With varied orbit and inclination, these systems offer tradeoffs between parameters such as pixel resolution, degree of global coverage, number of satellites, revisit time and mission life, etc. On the other hand, a vessel possesses many different physical properties by which it could be discriminated from other objects and/or background. Ship features include 3D-shape, size, texture, material composition, temperature variations and ship wake, etc. [5,6,7]. A range of different remote sensors could be designed which exploit some of these properties and help in identification. In [8,9,10,11,12,13,14] the authors have proposed the usage of high resolution panchromatic cameras to exploit ship size, shape and texture etc. While in [15,16] the authors exploited across-band analysis toward static/in-transit ships using a multispectral camera. Both these types of cameras could be used from airborne [7,17] as well as space-borne [18,19,20,21] systems during the daytime. These systems can provide very good accuracy in detection of ships and also enable classification to a certain extent of vessels into subcategories. The downside of such systems would be their limited swath (i.e., the image width captured by a camera in a single swipe). This makes it impractical for use as a scanner in vast oceans to identify all ships in any random point of time. Medium resolution systems proposed in [22] on the other hand provide wider swath, but suffer from relatively lower identification accuracy and cloud occlusions. In [23] authors proposed Convolution Neural Network (CNN)-based ship detection using high resolution satellite imagery and in [24] the authors have used ground-based CCTV video images to detect and predict the ship behavior. Both these methods have focused on inshore ship detection and behavior analysis near the sea shore, however these solutions are impractical for deep sea monitoring and larger region surveillance.

Active sensors such as SAR [2,6,25,26,27,28] -based imaging are more effective due to their all-time imaging and cloud penetration capability. However, these systems suffer from lower swath and infrequent revisit thereby resulting in the need for more satellites to cover the required region of interest. Though lower to medium Earth orbits provide semi-global coverage they have limitations related to revisit times, hence cannot provide images in continuous mode or at any time when required. Geosynchronous orbits are predominantly used for communication purposes, but recently there has been a growing interest among the user community in using them for imaging purposes too. Hence a system having coarser to medium resolution imaging capability, covering wider swath and higher revisit cycle would be an ideal solution for instantaneous detection of dynamic objects like a ship/vessel when the required region of interest spans a very large area.

Due to technological advancements in the last decade, the technique of on-board processing of satellite data [21,29,30,31] and information extraction is becoming a research hotspot. Researchers are looking into the feasibility of enhanced hardware-software blending for meeting end application needs. A complete processing chain for future intelligent observation satellites is presented in [30,31], where the authors proposed a fault- tolerant high performance computing system along with necessary storage. Although a real-time on-board ship detection method for an optical remote sensing satellite with 15 m resolution is proposed in [32], the implementations and results presented are for ground simulations only. Yao proposed [19] the use of COTS components to apply deep learning for ship detection for micro-nano satellites with a tradeoff between accuracy and model size. Yu proposed ship identification in the wavelet domain with Ostu’s threshold based on multi-scale salience enhancement [20]. Most recently, the Jilin-1 [18] satellite was flown with an onboard ship identification module for a 5 m resolution camera where the authors have used the aspect ratio parameter for achieving a higher degree of confidence. However, this method cannot be used for medium/coarse resolution images as the object occupancy in the image would be confined to one/few pixels.

This paper describes the uses of On-board Ship Detection (OSD) using a medium resolution panchromatic and/or multispectral sensor operating in NIR band, which can be placed on any spacecraft operating in Lower Earth Orbit (LEO)/Medium Earth Orbit (MEO)/Geosynchronous Earth Orbit (GEO). In medium resolution imagery, a ship would be manifested as a point object (or very few pixels) but has the advantage of wider swath covering more area in a single scan. Although ships can be identified using single spectral band (NIR having spectral bandwidth of 0.77–0.86 µm) carrying the desired signal, a multispectral camera operating in VNIR band (0.35–0.9 µm) with more than one spectral band would provide the added advantage of removing false identification, efficient land/water separation and motion/direction estimation. Identified geo-tagged points when used in conformity with the AIS data can provide an efficient ship/vessel monitoring capability and aid in identification of non-cooperative/non-confirming ships as it can negate the problems of AIS spoofing and switching off of AIS transceivers. The proposed system not only provides the information in real-time (or the order of milliseconds) but also uses negligible ground transmission bandwidth as only the results are transmitted to the ground. In conventional systems i.e., non-real-time systems the detection methods require the entire image to be formed, hence these systems either work on ground or require large amounts of onboard resources to perform the target detection process. The proposed system, designed for real-time and onboard implementation has an added advantage of working on a simple linear push broom imaging sensor with no requirements of substantial storage for staging the data or waiting for formation of complete image or even the requirement of an area sensor (imaging a complete frame). The entire solution is realized as an efficient pipelined architecture by considering all the stringent on-board constraints such as mass, power and resources. The rest of the paper is organized as follows: Section 2 describes real-time ship detection methods using a medium resolution optical camera. Section 3 details the design and architectural aspects. Section 4 outlines hardware implementation. Section 5 details the simulation results and analysis. Finally, Section 6 concludes with possible scope of future enhancements and directions.

## 2. Methodology: Real-Time Ship Detection

**Figure 1:**
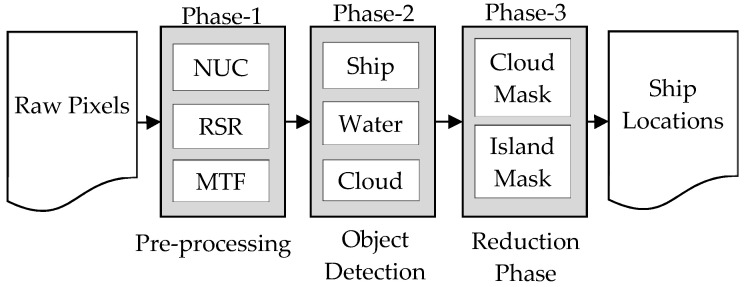
Ship Detection Methodology.

The process of ship detection is divided into three phases as shown in Figure 1. The first is a pre-processing phase, where the pixels undergo a sequence of enhancements to improve the radiometric quality of the raw signal received from the camera. The second phase is the object detection phase, during which the objects (in this context ships and ship like objects) are identified. The third phase is the reduction phase, where the falsely identified non-ship objects are eliminated based on contextual information (proximity criteria) to improve detection accuracy. Details are explained in the subsequent sections.

### 2.1. Pre-Processing

**Figure 2:**
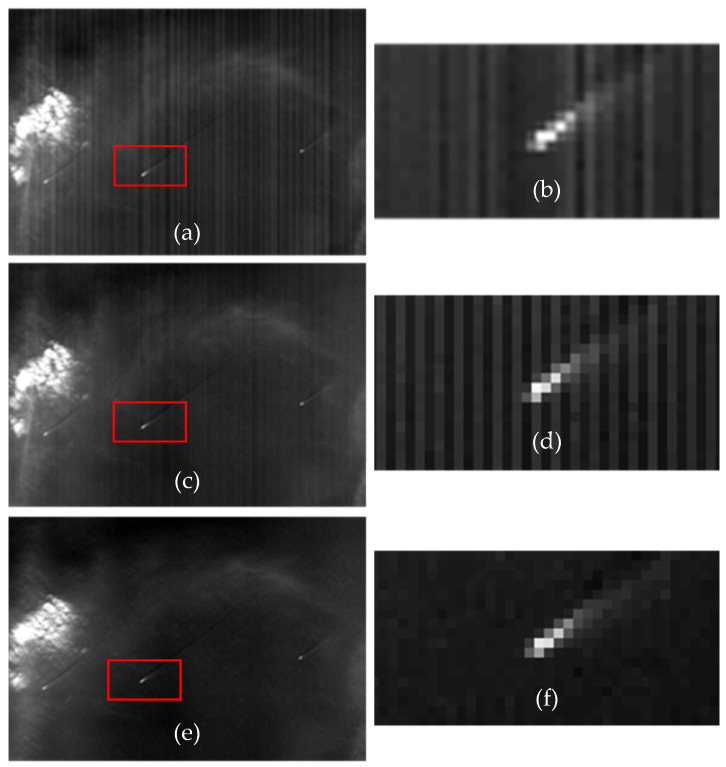
AWIF Image before and after preprocessing. (**a**) Raw Image with ship; (**b**) Zoom-8X of (**a**) Ship object; (**c**) Image after NUC applied; (**d**) Zoom-8X of (c); (**e**) Image after performing Residual Stripe Removal; and (**f**) Zoom-8X of (**e**).

Pre-processing, sometimes referred to as conditioning of the data, is an essential and crucial component in any object detection process. This phase consists of collection of image processing functions aimed at improving the radiometric fidelity so that the detection accuracy can be improved. The accuracy of detection is widely dependent on this phase as most of the detection algorithms demand good radiometric fidelity in images. The optical-electronics responses of the detector are deranged. This causes imbalances/artifacts in image radiometry including bands, stripe, blur, smear, vignette, ghost effect and port noise etc. This phase tries to minimize such imbalances by enhancing/restoring the image. Figure 2 depicts the outcome of a sequence of image processing steps performed on the raw signal data received from the camera electronic viz., detector Non-Uniformity Correction (NUC), Residual Stripe Removal (RSR) and Modulation Transfer Function (MTF) compensation. The steps are elaborated as below.

- Non-Uniformity Correction:

The responses of linear Charge Coupled Devices (CCDs) within a detector are not normalized, therefore it creates banding/striping artefacts in the image. The responses of the CCDs (referred to as light transfer characteristic or LTC) under different illumination levels are recorded often in the lab and the same are calibrated using validation sites after the launch. A Look-Up-Table (LUT) is made by applying the inversion of LTC and normalizing it across all the CCDs within the detector array. Although the derived LUT is non-linear in general, but in practice a best fit linear curve is derived and applied for simplicity [21,33,34]. The NUC correction followed in this article is as follows:

Let $X_{i}^{n}$ be the $i^{\mathrm{th}}$ raw pixel denoted in n-bit unsigned integer; and $G_{i}$ and $S_{i}$ be the corresponding gain and offset represented in signed fractional notation. Then NUC is described as in equation below:

(1) $$Y_{i}^{n}={\hat{S}}^{n}{(G}_{i}\times X_{i}^{n}+S_{i}),$$

(2) $$\hat{S}\left(X\right)=\{\begin{array}{cc} 2^{n}-1, & X\geq 2^{n}\\ X, & 2^{n}\geq X\geq 0\\ 0, & 0\geq X \end{array},$$

- Residual Stripe Removal:

NUC correction using Equations (1) and (2) should remove/balance most of the striping effects in the image. However, there may be scenarios where some amount of banding/striping artifact may be left in along-track direction i.e., the direction of satellite motion. This may be prominent in homogeneous regions including ocean waters. This artifact is due to linearization of NUC and hence residual stripe correction needs to be incorporated for efficient detection. The RSR compensates the remaining striping/banding artifacts by systematically analyzing and adjusting the radiometry in along-track (vertical or along the direction of satellite motion) and across-track (horizontal or perpendicular to the direction of satellite motion) directions. A common practice for normalization of satellite imagery using the image itself is based on the column averages [33]. Considering the real-time processing and on-board implementation, instead of using column averages of the entire image the RSR algorithm is modified for using a reasonable number of lines as explained below to achieve similar results.

Let ${\mathrm{DN}}_{x,\;y}$ be the digital number of an image I at position (x, y). Let $\mu_{\mathrm{al}}^{k}\left(x,\;y\right)$ and $\mu_{\mathrm{ac}}^{k}\left(x,\;y\right)$ be the DC estimation at point (x, y) in along-track and across-track direction correspondingly. In a direction k pixels are used to estimate the DC centered at (x, y). Then the RSR adjustment can be expressed mathematically as in Equation (3). Algorithm-1(a) explains the DC estimation used for on-board implementation. Algorithm-1(b) explains the overall steps for RSR:

(3) $$f^{k}\left(x,\;y\right)=\hat{S}\left({\mathrm{DN}}_{x,\;y}-\left(\mu_{\mathrm{al}}^{k}\left(x,\;y\right)-\mu_{\mathrm{ac}}^{k}\left(x,\;y\right)\right)\right)$$

**Algorithm 1.** (a) DC signal $\mu_{*}^{k}$ Estimation and (b) RSR correction.(a)Input: DN[N] be the array of N pixels and n is the percentage of noisy pixels that to be removed before computation. Let m be the method of estimation; here two methods are envisaged viz. mean or median.$\mathrm{Step}\;1:\;\mathrm{sort}\;\mathrm{the}\;\mathrm{buffer}\;{\mathrm{DN}}_{\mathrm{sort}}\left[N\right]$ from DN[N];Step 2:  if(m==median)                      ${\mathrm{Res}=\mathrm{DN}}_{\mathrm{sort}}\left[N/2\right];$
        **Else if**
(m==Mean)
Step 3:   Sum=0;
       
$N_{\mathrm{noisy}}=\frac{\left(n*N\right)}{100};$

     **For**
${i=N}_{\mathrm{noisy}}\;\mathrm{to}\;\left({N-N}_{\mathrm{noisy}}\right)$
           ${\mathrm{Sum}+=\mathrm{DN}}_{\mathrm{sort}}\left[i\right];$
$R\mathrm{es}=\mathrm{Sum}/\left({N-2*N}_{\mathrm{noisy}}\right)$(b)Input: ${\mathrm{DN}}_{x,\;y}$ be the pixel of image I[X, Y], d is the depth of the window, n is the percentage of noisy pixels and m be the method;Step 1:  **For** all the pixels x in along track do
Step 2:    **For** all the pixels y in across track do
Step 3:      **For** i=-d to d
**do**          **if**
0≤(x+d)≤X then set,              $\mathrm{Col}\left[i+d\right]{=\mathrm{DN}}_{x+d,\;y}$;      **else** set Col[i+d]=0;Step 4:      ${\mathrm{DC}}_{C}\left[y\right]=\mathrm{EstDC}\;\left(\mathrm{Col},\;2d+1,\;n,\;m\right);$
Step 5:    **For**
all the pixels’ y in across track do
Step 6:       **For** i=-d to d
**do**          **If**
0≤(y+d)≤Y then set          1.   $\mathrm{Row}\left[i+d\right]{=\mathrm{DC}}_{C}\left[y+d\right];$
          else set Row[i+d]=0;Step 7:    ${\mathrm{DC}}_{R}[y]=\mathrm{EstDC}\left(\mathrm{Row},\;2d+1,\;n,\;m\right);$
Step 8:    **For** all the pixels y in across track
**do**           $\mathrm{tmp}=\left({\mathrm{DC}}_{R}\left[y\right]-{\mathrm{DC}}_{C}\left[y\right]\right)$;$\mathrm{RSR}\left[x,\;y\right]{=\mathrm{DN}}_{x,\;y}-\;\mathrm{tmp}$;

- Modulation Transfer Function:

Due to inaccuracies in the optics manufacturing, atmospheric effect and motion of the satellite etc. the image seen by a camera system gets blurred. To correct this blur, a restoration process is applied, which is also referred to as MTF compensation. Often the compensation is estimated using a Point Spread Function (PSF) and is computed based on a point source within an image or by imaging a distant star.

Let image g be the image obtained from an imaging system, with h as PSF, f as clear image and w is the amount of noise; then MTF is modeled as in Equation (4). Most often restoration is done in frequency domain, by applying the Fourier Transform. In frequency domain the model is explained as in Equation (5). Again considering the on-board real-time processing requirement an equivalent kernel in spatial domain is generated for instantaneous operations. Let C_2k+1, 2k+1_ be the equivalent coefficients that are generated to perform PSF in spatial domain and X_m, n_ be the image pixel at position (m, n) then, the convolution operation is denoted as in Equation (6):

(4) g(m, n)=h[m, n]×f(m, n)+w[m, n]

(5) $$\hat{F}\left(u,\;v\right)=\frac{G(u,\;v)}{H(u,\;v)}=\frac{H\left(u,v\right)F(u,\;v)}{H(u,\;v)}+\frac{W(u,v)}{H(u,\;v)}$$

(6) $$f\left(m,\;n\right)=\hat{S}\left(\sum_{i=-k}^{i=k}\left(\sum_{j=-k}^{j=k}\left(C_{k+i,\;k+j}{\;\times \;X}_{m+i,\;n+j}\right)\right)\right)$$

### 2.2. Point Object Detection

In a medium resolution optical sensor, ships appear as point objects with a few pixels spread. To identify and discriminate ships from background clutter, land-water separation, cloud identification, and cirrocumulus cloud identification are also essential. This phase consists of two independent sub tasks. The first sub-task is identification of ship-like objects, for which a variant of CFAR [25,26,28,35] is applied. The second sub-task is a tradeoff between two strategies viz., min-max threshold and Normalized Difference Water Index (NDWI) [36] for land-water separation. The dense and cirrocumulus cloud identification are addressed in reduction phase.

**Figure 3:**
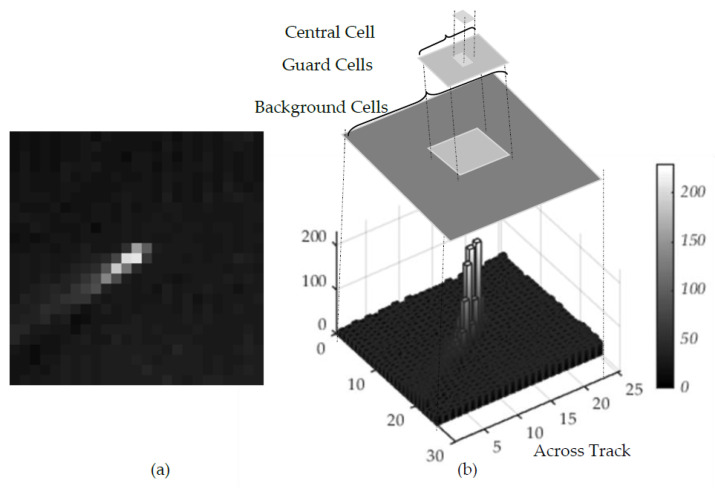
CFAR (**a**) Pre-processed Image with ship; and (**b**) 3D-barchart of the Image.

- Constant False Alarm Rate:

In the VNIR band, the reflectance of metallic components is relatively higher than that of the water. Figure 3a shows the DN values of ship and background water. The DN value of the ship is higher and appears relatively brighter than its background which is water. The CFAR analyzes and thresholds such localized area. As shown in Figure 3b, a localized 2D-block of pixels is partitioned into three groups referred as central cell, guard cells and background cells. Central cells or target signal cells are the area where the reflectance is highest in the VNIR band (due to the metallic body of the ship) and the background is the calm watery area where the reflectance is minimal. A ship is declared to be positive if the central cell is distinguishable, i.e., central cells signal is substantially greater than that of its background. Guard cells are the area or cells in between the central cells and background cells. The cells where the signature of background cells and central cells overlap, thus we obtain mixed response of object and the background cells. Often, it’s existence as background increases background power estimation and reduces object detectability [25]. During our simulation, up to two contours of cells outside of central cell are kept as guard cells or forbidden cells to avoid such interference. The overall window size is kept as configurable up to 9 × 9.

Additionally, a few more constraints are checked in the localized area before positive identification is confirmed. This includes parameters like min-max DN threshold for a valid ship pixel in central cell, minimum ship pixel occupancy in the guard cells and minimum water occupancy in the background cells. These additional constraints reduce false identification significantly.

Let ${\mathrm{DC}}_{\mathrm{cc}}$ and ${\mathrm{DC}}_{\mathrm{bc}}$ be the mean signals of the central cell and background cells correspondingly, and ${\mathrm{SD}}_{\mathrm{bc}}$ be the standard deviation of background signal. A constant factor α also referred to as false alarm rate is fixed such that, Equation (7) satisfies the point to be potential ship. The threshold α plays an important role in deciding the tradeoff between false alarm and missed detection. A small value of α would increase false identification, while a very high value leads to failed detection. In our implementation, the range of α in between 2.0 to 10.0 is considered as optimal region for threshold. Further, to avoid blunders caused due to the imaging system such as pixel dropouts, degraded pixels and background clutter etc.; noisy pixels are removed by ignoring the minimal and maximal pixels in the background cells while computing statistics about background:

(7) $$\left({\mathrm{DC}}_{\mathrm{cc}}{\;-\;\mathrm{DC}}_{\mathrm{bc}}\right)\;\geq \;\alpha \;\times \;SD_{bc}$$

- Land-Water Separation

**Figure 4:**
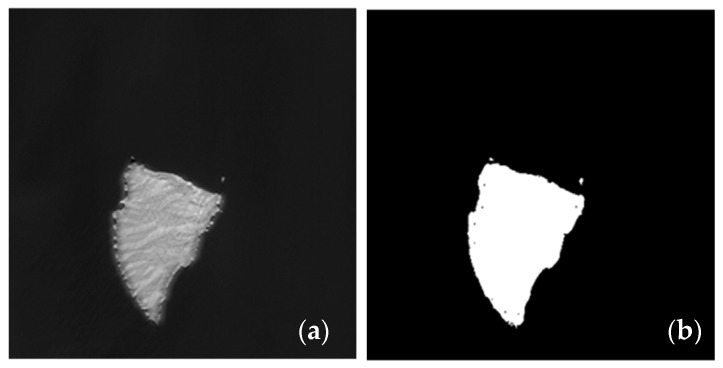
Land/Water Separation (**a**) Before separation; and (**b**) After Separation.

To strengthen the detection accuracy a few more constraints are added. The first constraint applied is to allow CFAR to detect only in water as background. This results in the separation of land and water in the images. Two strategies are adopted to separate water from land. In the simplest form segmentation is used by creating max-min threshold derived from an empirical model using single NIR band alone. Figure 4 depicts a threshold based empirical model on single NIR band. Alternatively, using two bands (typically green and NIR spectrum) methods like NDWI [36] may be applied to identify water bodies accurately. We have chosen single band simple threshold implementation for separating land and water, however one can also use NDWI approach using two band data. The NDWI for land water separation can be implemented as explained below.

Let ${\mathrm{DN}}_{g}$ and ${\mathrm{DN}}_{n}$ be the digital number under green and near infrared spectrum correspondingly within a cell, then a normalized ratio is computed as in Equation (8). A positive ratio indicates non-water, while a negative number indicates water:

(8) $$\mathrm{NDWI}=\left(\left({\mathrm{DN}}_{g}-{\mathrm{DN}}_{n}\right)/\left({\mathrm{DN}}_{g}{+\mathrm{DN}}_{n}\right)\right)$$

### 2.3. Reduction Phase

False alarms are an integral part of any object detection process. Hence, the detection mode is always augmented with reduction process/phase to minimize false detection. In the case of ship like object detection from medium to coarse resolution imagery, cirrocumulus clouds can cause a lot of false alarms as the signatures of cirrocumulus clouds are similar to those of ships. The whole process of reduction is done in two parts, viz., masking of densely spread cloud and discriminating the sparse point cloud toward cloud edges which are not identified by the first step. Both the portions are masked as cloud and the ships identified (if any) under the masked regions are not considered and are simply ignored.

**Figure 5:**
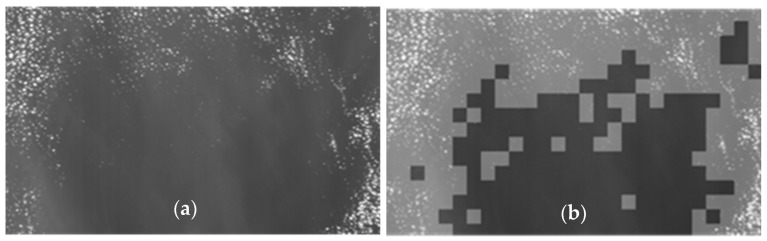
Cloud Mask (**a**) Original Image; and (**b**) After Cloud mask.

For densely spread cloud masking, the strategy adopted is block-threshold. A suitable tile size and a threshold τ is derived using an empirical model for identification purpose. Let $I_{x,y}$ be a tile of image with standard deviation $\sigma_{x,y}$ then ${\mathrm{Cloud}}_{x,y}$ is computed using Equation (9). A positive notion indicates the tile is cloudy and is masked. There can be scenario, where a tile is partially covered by cloud (typically happens toward cloud outer periphery) and the tile is not masked. This happens due to tile-by-tile processing. To avoid such issues, an additional tile adjacent to the outer fringe of cloud can be masked as shown in Figure 5.

(9) $${\mathrm{Cloud}}_{x,\;y}={\tau -\sigma }_{x,y}$$

The second part is masking sparsely distributed cirrocumulus clouds which were left unmasked in the previous step and have very similar spatial and radiance values to ships but occur in groups. On the other hand, ships follow a safe distance while in motion hence are relatively sparse except in pre-known static areas such as docking areas. These pre-known areas can be geo-fenced to avoid misidentification. In the remaining area, clustering property of thin clouds viz., its spread and cardinality can be exploited (as explained below) to discriminate from the ships.

Let ρ (> safe distance) be the radius of a circle with ship under inspection in its center and η be the total number of detections within the specified area. Let κ be the maximum number of ships that can exist practically in motion within the circle, and can be derived by an empirical model or a-priori knowledge of safe ship distance in the sea. Then, η>κ indicates a false identification which can be discarded. In our implementation the values are configurable. For simulation purpose ρ was fixed at 64 pixels and κ having value more than six ships are masked for non-consideration.

## 3. Design and Architecture

**Figure 6:**
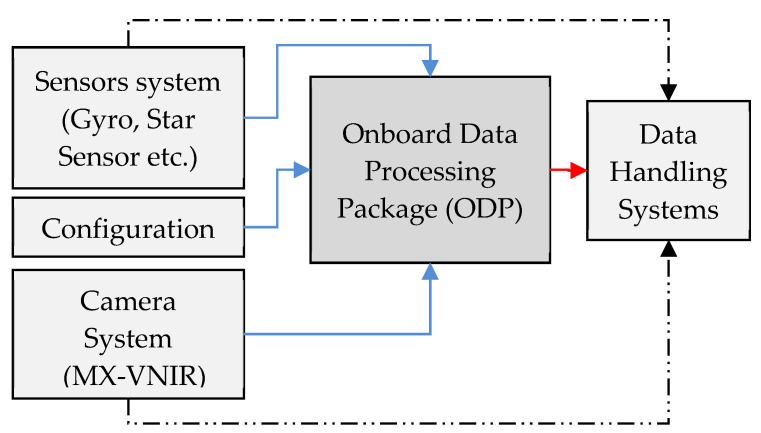
ODP Package Realization plan.

The design objective is to identify ships from onboard a spacecraft. The objective is also influenced by non-functional goals such as execution timeline, reliability and availability which are very crucial for any early warning system. The other goals related to any on-board implementation include resource optimization and scalability/configurability for the future. Realization of the proposed on-board ship detection is envisaged as one single package namely an On-board Data Processing (ODP) package. The overall scenario and connectivity with other subsystem is shown in Figure 6. The dotted black lines indicate the setup where no ODP package is placed and the data gets transferred to ground directly via data handling systems without a ship identification process in-loop. In the new configuration, the ODP package is added in between and it receives input from the MX-VNIR camera system, ephemeris data from sensor systems such as Attitude and Orbit Control Systems (AOCS) and produces an output packet consisting of ship locations. The output packet is forwarded to data handling system which is shown in red color. The detailed design and implementation are discussed in the following sections.

### 3.1. Hardware Selection and Scalability

Considering on-board implementation, two options emerge as viable solutions viz., Application-Specific Integrated Circuits (ASIC) and FPGA. FPGA is chosen primarily for the ability to reprogram for a prototype design, relatively shorter development time and most importantly for fewer numbers of installations. On the other hand, modern day FPGAs are packed with high density macro blocks making it suitable to cater to bigger designs with higher frequency. For the current mission, the package is realized using the radiation-hardened Virtex-5 series of FPGA with part number-XQR5VFX130 as the processing platform from Xilinx, Inc. (San Jose, CA, USA). The implementation level challenges to realize all the algorithms mentioned in Section 2 on FPGA are discussed in the subsections below. The design is kept as generic as possible with two layers of scalability in mind. At the logical layer, the hardware definition, firmware and software can be upgraded with enhanced functionalities during mission life and at hardware layer, newer components may be mounted to cater for future missions’ incremental requirements.

### 3.2. Design Simplification and Packing

Although FPGAs greatly reduce the engineering and financial burden, the architecture is still expensive in terms of resource utilization, especially for deep logic such as division, square root, logarithm, power, trigonometry functions etc. Most often Volder’s algorithms are used to deal with such problems, where tradeoffs are made between accuracy and latency. To deal with these two strategies are adopted—algorithm simplification and step simplification. First, the algorithms are chosen such that they are amenable to FPGA architecture (ex. recurrence/loop-free steps). Second, the steps are modified to reduce unnecessary complex logic (ex. instead of standard deviation variance can be computed avoiding square root operations). Further, a low latency sorting technique such as bitonic sort [37] is adopted for the problem like sorting, rank-ordering etc. An equivalency test is essential for verification about correctness of result with respect to the modifications.

Unlike conventional buffer-based designs where the design requires a huge off-chip memory for buffering the entire image, our design is made for streaming data with push-broom imaging sensors. This means that the package is directly connected to the camera electronics with streaming data recorded line by line and is processed on-the-fly with minimum latency.

In current technology, almost all the FPGAs are packed with DSP48 blocks; these blocks are extensively used for implementing fixed point multiplication/addition operations. This simplifies the design by reducing the burden on slices. Further FPGAs of current technology are fused with enough on-chip memory modules such as block-RAM, etc., to satisfy the need of intermediate line buffering. However, in our design off-chip memory is also provisioned for storing the configuration parameters and also for the purpose of aligning the pixels across bands in two band configuration.

### 3.3. Resource vs. Accuracy

**Figure 7:**
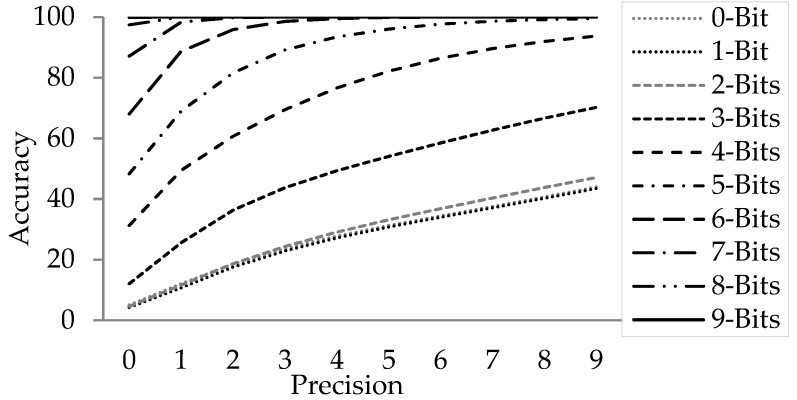
Accuracy against Bit-Precision.

The resource requirement related to deep logical operator becomes worse in the presence of floating point arithmetic (standard IEEE-754). It has two shortcomings—firstly suboptimal usage of silicon due to standard driven requirement instead of application driven. Secondly, unwanted packing/unpacking of bits builds up the system latency. To avoid such limitations a relatively relaxed data type—fixed point notation is used. In this notation, integer arithmetic is used to manipulate different operations. Although due to the fixed point arithmetic there would be loss of precision thereby resulting in inaccuracies. A careful analysis is done at every step and only reasonable number of fixed point bits are used for computation so that the end result is not compromised. Figure 7 shows the achievable near-float resultant accuracy at various bit-depth of fixed point computation for the method explained in Section 2 with our test data. The various dotted lines represent the bit-depth used for fixed point computation. X axis represent the number of bit used for comparing the fixed point to float output and Y axis represent cumulative percentage of pixels that are same as floating point operation at the given precision. This design achieved better than 97% accuracy with 8-bit fractional computation. Thus, the system is developed with 16-bit signed fractional data type representation with 1-bit for sign, 7-bits for the mantissa and 8-bits for the fractional part.

### 3.4. Configuration Based Execution

The system is designed to be very generic and suitable for use with a wide variety of sensors. Sensor specific parameters are kept configurable to abstract from the implementation. The configurability accounts for many sensor dependent behavior as well as algorithm tuning during mission life. Specifically, degradation of detectors which can happen during the mission life, algorithm parameters such as cell size, false alarm rate and various thresholds etc., are kept as configurable and are generated empirically using the actual data.

Based on frequency of operation, two types of configuration are envisaged—TYPE-1 and TYPE-2. In TYPE-1 configuration, controls are reflected immediately and are mostly Boolean in nature such as enabling/disabling certain modules and mode-of-operation etc. Total 8-bit TYPE-1 configuration is envisaged. On the other hand, a total of 512 bytes of TYPE-2 command is generated to control module specific resistor such as constants, coefficients and thresholds. Both the configurations are packed in the form of a command by configuration coder (software) on ground and can be uploaded in part or full through tele-command to ODP package. On-board command decoder decodes the data before using the configuration.

## 4. Implementation

**Figure 8:**
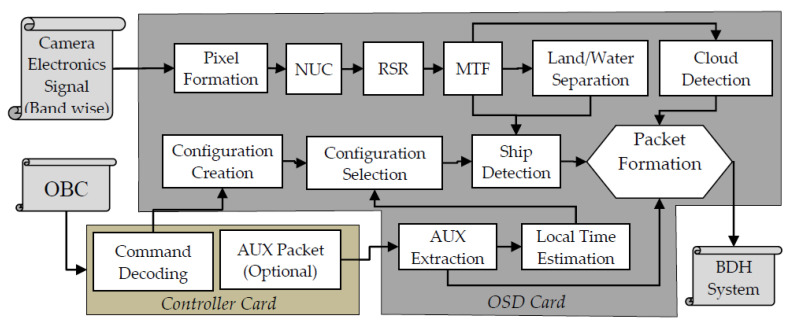
Block Level Detail Design.

The package designed and implemented for this purpose is referred as ODP. As shown in Figure 8, the package consists of interface with other subsystem and two cards —a core card and a controller card. The core card is responsible for processing of all the Algorithmic core and is referred to as OSD card. The controller card is responsible for power management and interfacing with other subsystems.

Primarily, the package has three input interfaces and one output interface. The output is sent to the Baseband Data Handling (BDH) system for transmission to the ground. The input interfaces for the OSD package consist of:

○ One/two bands of camera signal from the camera electronics (CE). NIR may be used as primary camera and green can be used as secondary band in two band scenario. A push broom camera having up to 16K CCD elements with up to 12-bit pixel depth can be connected with the package through this interface. The current OSD package is implemented for an upcoming satellite having a push broom camera with six bands in MX-VNIR each having a detector array of 12K CCD elements with 12-bit pixel depth and 42m spatial resolution.
○ The package receives Ancillary (AUX) data pertaining to orbit and attitude sensor, UTC time and modes etc. from the on-board computer (OBC) through a 1553 IP interface.
○ Command and control for configuring and health monitoring of the package is done through a 1553 IP interface with OBC.

The OSD core card receives camera signals from CE, commands from the command decoding module and required AUX information from the controller card. Once all the bits for a line are received by the core card, pixel formation takes place. Then pixels of the line are forwarded to improve radiometric fidelity including NUC, RSR, MTF. Subsequently, the corrected line is given as input for land/water separation, ship detection, cloud detection in parallel to the respective modules. Finally, the ship results go for packet formation before passing to BDH. All the modules maintain an internal buffer for holding few image lines (window depth) so that every time a line of image is given as input to any module it produces one output line about its window-center. In case of block based modules such as Cloud detection, results are updated after the last image line of the block in acquired/processed.

The controller card interfaces with the onboard computer and OSD card. The controller card consists of 1553 IP core and 8086 IP core. Through the 1553 interface of controller card it receives all the commands from ground pertaining to OSD. A command decoder module extracts the commands and generate appropriate signal for OSD to function. This card also receives AUX packets from OBC and forwards relevant portions to the OSD card for the purpose of geo-referencing.

Two modes of configuration selections are envisaged: auto and manual. In auto mode, the AUX extraction module extracts relevant fields, including time tagged orbital position, look angle etc., and passes it to the Local Time Estimation (LTE) module. This estimation helps in appropriate selection of a configuration to account for the Sun illumination angle during different times of a day. If manual mode is selected, the configuration is uploaded using ground command. Further, this module provides the result in a bitmap about presence and absence of a ship, with a logical high value indicating the pixel to be a ship; while a logical low indicates the pixel not to be a ship. The exact positional information in georeferenced coordinates (latitude and longitude) is derived on ground using the orbit and attitude modeling from the star sensor, gyro, etc. To meet this criterion relevant fields from AUX are also packed in the OSD output packet. In future the coordinates are planned to be computed and sent without the bitmaps.

For every input line, the OSD generates an output packet compliant to the standard. All the output signals generated for BDH system is synchronous with respect to line pulse. Primarily, the output packet consists of three major fields viz., ship, cloud and ancillary information apart from the packet header. Although the design can handle up to 16,384 pixels, the envisaged mission only has 12K pixels. Hence, to reduce the transmission bandwidth further a relatively smaller size (2400 bytes) packet is constructed by the packet formatter. An area of size 1500 bytes (≡12,000 bits) holds the binary decision about a pixel to be a potential ship; another 24 bytes area holds the information about cloud and 876 bytes are reserved for holding the AUX information. Under inspection/debug mode of operation, 1500 bytes of ship area is reused with appropriate flag where the intermediate results of 1200 pixels (@10 bit ≡12,000 bits) are packed and transmitted to ground for further analysis.

Based on neighboring pixel dependency, the image operations are segregated into three categories, viz., point, windows and block operations. In the first category, a pixel operation does not depend on the neighboring pixels. These are simplest to implement and impose only a few clock delays. The modules such as NUC, land/water separation, etc. fall under this category. The second category is about cell/window operations, which are relatively heavy in terms of compute complexity and resource requirement. These incur constant amount of along-track latency for buffering/availability of real-time data. Modules such as RSR, MTF compensation and CFAR etc. fall under this category. This translates into more delays as until the required number of pixels/scans are available, the processes would not start thereby resulting in pipeline latency and shift in the output in spatial domain. In block processing the operator is applied on the entire block of data to achieve a single result, unlike point/window operations where output is generated corresponding to every pixel. Typically, block size being more than window size, buffering requirement as well as latency is higher. Modules such as cloud detection fall in this category. Table 1 below summarize all the operations in OSD package along with the problem size, pipeline delay or line-shift in along-track direction, buffer requirement and incurred latency of the implementations in terms of clock ticks.

In general, the clock frequency requirement by pipelined systems are less as compared to time-slice driven processing systems (such as CPUs) and are power efficient. The design of this order, proposed in this paper can be realized by modern FPGAs with the required clock frequency. For the purpose of a simpler design the entire design is kept under a common clock of 32 MHz. The overall latency of the system that is from first-in-to-first-out is observed in the order of a few milliseconds. The present version implementation contains single band design scenario. The design was implemented on a Xilinx radiation-hardened Virtex-5 series of FPGA with part number-XQR5VFX130. The resource utilization summary is given in Table 2.

During various phases of development, a wide set of COTS/custom built hardware was used. The Xilinx ML605 development kit was used for module-level development and testing. The integration of the overall development was carried out and functionalities/performances were verified using a custom built ODP hardware (commonly known as engineering model) with industrial grade components. Finally, a Flight Model (FM) was realized with space grade components equivalent to the engineering model. The FM was subjected to different environmental hazards such as thermal shock, vacuum, vibration, ionized environment, etc., and stability in outcome was monitored and qualified. The highlights of package parameters for the custom-built package are tabulated in Table 3. The tools that were used during various analysis, development and testing phases are tabulated in Table 4.

## 5. Simulation Results and Analysis

### 5.1. Dataset Preparation

In order to verify the effectiveness of the system designed the dataset is prepared using raw signal from AWiFS camera of Resourcesat-2/2A. The AWiFS’s technical highlights are tabulated in Table 5 below. The dataset consists of moving and parked ships with different dynamics including calm ocean bed, clutter sea, near coast line, deep sea and docking areas. Further, to assess the robustness of the methods in terms of wrong-identification and misidentification, negative conditioned data is also supplemented in the test data set. This includes dynamics such as seasonal variance, wide range of clouds, fog interference and small islands etc. band B4 (NIR band) is chosen as the primary band for ship identification. In the event of two-band processing band B2 (Green) serves as secondary band.

### 5.2. Configurations Creations

In any object detection method, detection capability is attributed primarily by two matrices—recall and precision. Formally, recall and precision are defined as in Equations (8) and (9). Thus recall is the ability of a system to detect all the valid objects whereas precision is the truthfulness of a system’s output. Our system can be configured for a wide range of applications by tuning its control parameters to meet the end application need. For example, in search-&-rescue mode of operations the system may be constrained to produce high recall with little degradation of precision, whereas for daily patrolling purposes of the same area false identifications may be minimized and system can be biased toward higher precision. The list of configuration parameters during detection and reduction phases along with their definitions are tabulated below in Table 6. All the threshold parameters are set statistically, based on the application in mind and a suitable configuration is formulated. To set the minimum/maximum threshold of a parameter appropriate data set is selected and is computed by Equation (10):

(10) Threshold=μ ± n × σ

where μ and σ are the mean and standard deviation of the configuration parameter’s distribution present in the data set. For a given application in mind, relatively lower factor n makes the configuration tighter and improves precision at the cost of recall, whereas a higher value relaxes the configuration and thus improves recall at the cost of false alarms.

### 5.3. Test Setup

**Figure 9:**
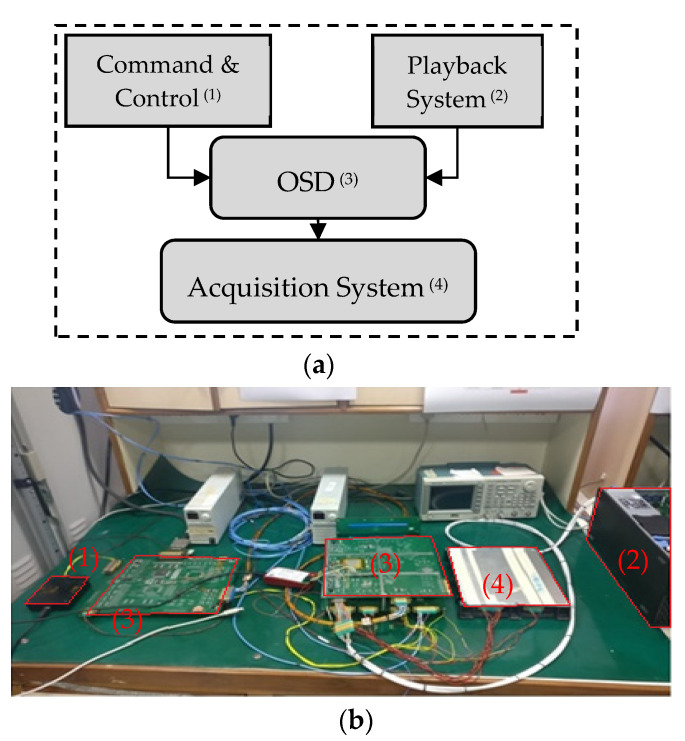
Simulation Setup (**a**) Test setup Block Diagram (**b**) Annotated EM Model Simulation.

As depicted in Figure 9 below, the test setup consists of four sub-systems and are annotated in red color numbers, viz., playback system ^(1)^, command and control ^(2)^, the OSD ^(3)^ package and acquisition system ^(4)^. The playback system or data simulator mimics the camera electronics and can restream an already acquired image with appropriate interface definition. The second is a command and control simulator which translates the configuration into a command and uploads the command to the package. The third one is the OSD package itself where based on the configuration, processing/ship identification is performed and the source packets are formed and forwarded. Finally, an acquisition system, receives the packets as per data-handling protocol, unpacks and analyzes the results for debug, analysis, and reconfigurations purposes.

### 5.4. Detection Analysis

In this sub-section the effectiveness of our method is summarized. To evaluate the system behavior quantitatively, standard statistical metrics precision and recall are used as discussed earlier. R is recall, P is precision, n is the number of ship correctly identified by the system, A is the actual total ships that are present in the data set and D is the total ships that are identified by the system. Then the precision and recall are defined to be:

(11) $$R=\frac{n}{A}\times 100\%$$

(12) $$P=\frac{n}{D}\times 100\%$$

In order to quantify the system’s ability to identify ships statistically, a total of 10 strips of data were used as test bed and corresponding true ship positions were identified manually by human operator using eye-ball technique (as the corresponding AIS data were not available due to random acquisition). To reduce human identification error 3-operators were assigned the same task independently and majority voting was applied. For the system to identify ships in these data sets a general configuration was prepared at n=2.5 using Equation (10).

**Figure 10:**
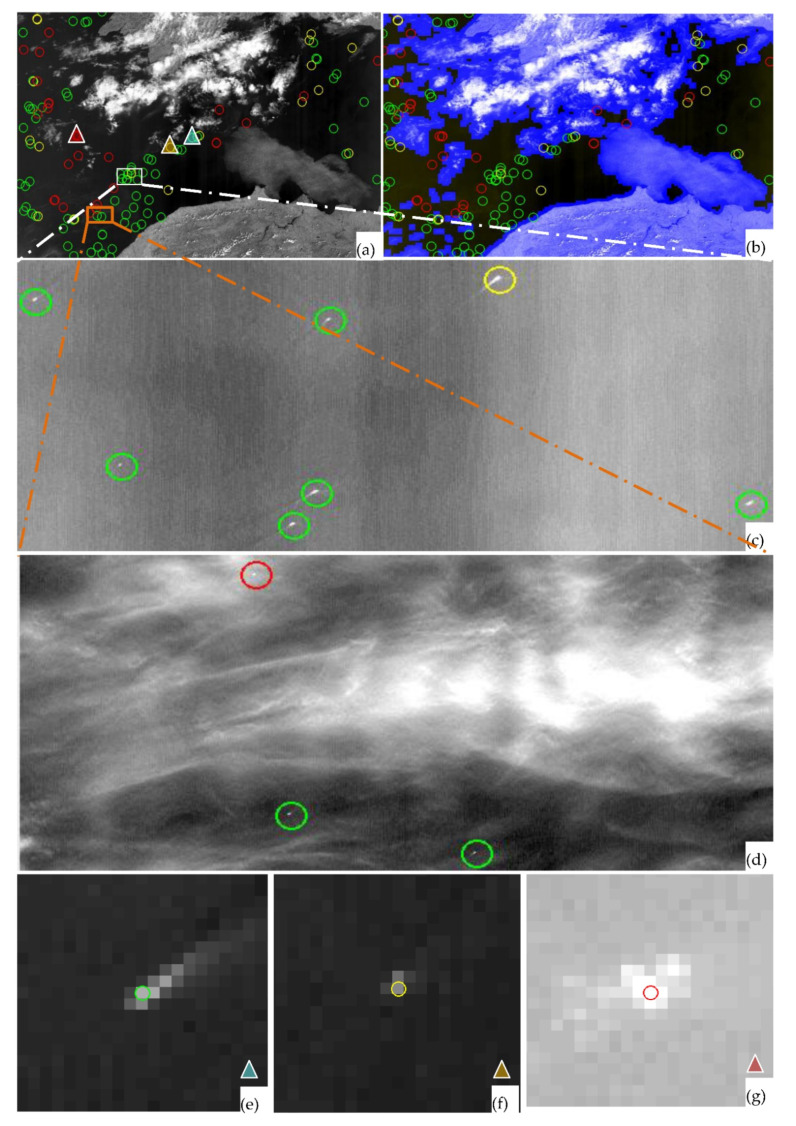
(**a**) Ship Detection in scene-2: Green: Identified, Yellow: Missed, Red: False (**b**) After cloud mask (**c**) Nominal Identification (**d**) Identification in Cloudy region (**e**) Identified (**f**) Missed (**g**) False scenario.

Table 7 tabulates the scene-wise statistics. Column-2 specifies ships counts that were identified by the human operator. Column-3 shows the ships identified by our package for the same scene. Column-4 indicates true identification of ships by the system in-line with the human operator. Column-5 indicates the number of ships that are identified by the operator which the system could not, while column-6 indicates the number of ships that are identified by system but not in conformity with the human operator i.e., false-identification. The last two columns indicate precision and recall percentages rounded off to the nearest integer (as defined in Equations (11) and (12)). With no specific configuration in mind, the overall precision and recall achieved is 64% and 86%, correspondingly. For better visual understanding part of scene-2 is extracted and shown in Figure 10a, where circles in green, yellow and red indicate identified, unidentified ships and false alarms by the package. Figure 10b shows the non-water/cloud region as blue-mask. Figure 10c,d shows calm and cloudy region detection capabilities correspondingly while Figure 10e–g show zoomed outcomes.

**Figure 11:**
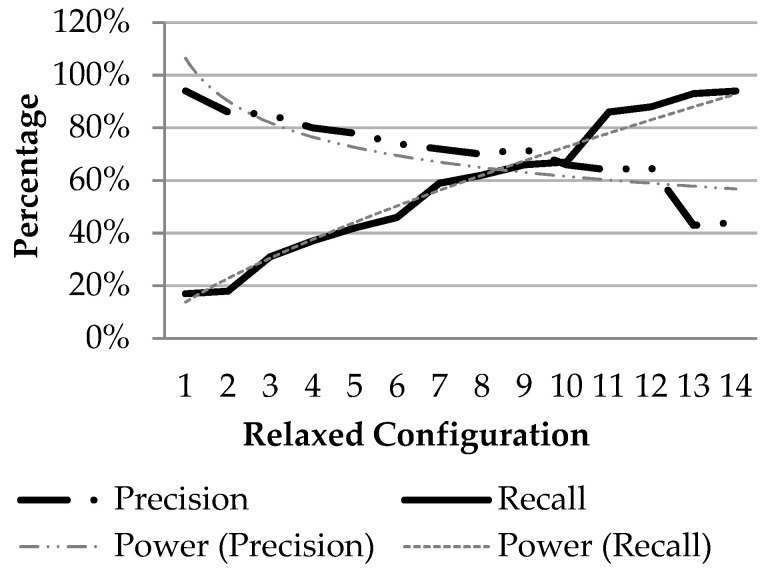
Precision vs. recall graph.

To analyze the system behavior under different applications a set of 14 different configurations were prepared. The analysis is plotted in Figure 11, where *X*-axis indicates different configuration with relaxed parameters, while *Y*-axis indicates the percentage. With relaxed configuration recall improves gradually but precision goes down. The corresponding thinner graph indicates the trend of the graph is logarithmic. The behavior of tradeoffs between recall and precision is as expected. The plot suggests the first five configurations may be used for higher precision applications for example: day-to-day patrolling, the last size five configurations may be used for high recall applications such as search-&-rescue and the middle four configurations could be used where precision and recall are required to be unbiased.

## 6. Conclusions

Ship detection is an important aspect of maritime surveillance. In this paper a method for implementing ship detection onboard a medium resolution satellite is presented. A three stage methodology is used for ship-like object detection. In the first stage the raw data from the camera is pre-processed to enhance the radiometric fidelity using of host of algorithms like NUC correction, stripe removal, etc. In the second stage a modified CFAR algorithm is used for detecting ship-like objects. The parameters used such as threshold values are fixed based on heuristics and empirical studies. Later in the third stage, i.e., reduction stage, false alarms are removed/masked, which are mainly caused due to the presence of clouds in the data.

The key advantages of the realization include: (a) the methods suggested are amicable for implementation in a resource constrained environment such as the onboard system, real-time systems, (b) without any loss of generality the proposed preprocessing methods are applicable for any optical sensor for the purpose of radiometric correction and enhancement and (c) a buffer-free streaming design was adopted by avoiding the need for off-chip buffers, which is ideally suited for any real-time application, 24 × 7 seamless operations and most importantly, onboard satellite implementation.

The ODP package was realized using two cards, viz., an OSD card and a controller card, all using space-qualified components and subjected to space qualification processes. For testing an environment was created and existing satellite data closer to the specifications of the sensor to be flown, i.e., 56 m resolution AWiFS data in the spectral band of 0.71–0.74 µm pertaining to IRS-Resourcesat-2 was played back and received by the ODP hardware package. The output was received and verified in the lab. The results were quite encouraging and satisfactory. We could achieve a best precision result of 64% and a recall of 86% for most of the scenes. If we further dwell on the fact the results in clear waters are much better than the above observation.

In the future we propose to use multiple bands to improve the accuracy and also incorporate geocoordinate generation. We also intend to extend the same methodology for other objects/events such as the detection of forest fires, anomaly detections, etc., using similar sensors or other resource constrained missions like micro/nano satellites. Another line of work is towards extending it to other sensors like SAR and high resolution MX/PAN for different applications. Autonomous processing can also be attempted to enable the system to be self-reliant, which can be achieved by learning from the data itself. This also minimizes the ground command uplink. Other areas of work are towards extending it for onboard computing and feature detection strategies for smart sensing, swarm or constellation flights for collaborative/autonomous imaging and decision making.

## Figures and Tables

**Table 1:** Latency Aspects.

	Operation (Category)	Window Dimensions	Buffer Size (kilo pix)	Clock Tick	Spatial Shift
NUC	Point	1		28	0 lines
RSR	Window	Up to 15 × 15	16 × 16	134	9 lines
MTF	Window	Up to 7 × 7	8 × 16	72	5 lines
CFAR	Window	7 × 7 and 9 × 9	10 × 16	112	6 lines
Land/Water Separation	Point	1	-	-	0 lines
Cloud Masking	Block	64 × 64	188 (int_64)	32	64 lines

**Table 2:** Resource Utilization Summary.

XQR5VFX130	Used	Available	% of Utilization
LUT	47,250	81,920	57%
FF	41,387	81,920	50%
Block RAM	213	298	71%
DSP48	202	320	63%
IOB	32	836	3%

**Table 3:** (**a**) ODP-10 Package Parameter, (**b**) ODP-10 package, OSD card and controller cards

Package Name	ODP-10
Input Port	VNIR- LVDS Serial OBC-MIL-STD-1553B
Output Port	DHE-LVDS Serial
Weight	5.0 kg
Size	341 × 273 × 158 mm
Power	8.4 W
Processing Time	16 line duration
(a)	
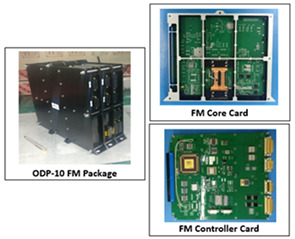	
(b)	

**Table 4:** Development and Verification Environment.

Purpose	Package/Software/Utility
Software Simulation	C, C++
Design Entry	VHDL-2002 and Handle-C cross compiler
Design Simulation	Questa simulator from Mentor Graphics
Synthesis & Implementation	Xilinx ISE13.2 with sirf overlay utility package
Visual Inspection and Analysis tool	AIPS-viewer (In-house developed viewing software)

**Table 5:** AWiFS Specification.

Camera Name	AWiFS
Imaging Type	Pushbroom Imaging
Number of Cameras	2; (AWiFS-A and AWiFS-B)
Pixels	6000 Pixels/Detector; Total 12,000
IGFOV	56 mtr @nadir
Swath	740 Km with 2 Cameras
Spectral Bands	B2: 0.52–0.59, B3: 0.62–0.66, B4: 0.77–0.86, B5: 1.55–1.70
Quantization	10 bit with DPCM or MLG

**Table 6:** Parameter definitions

Detection Phase Configurations	
Parameter	Definition
Min Alpha	Minimum False Alarm Rate related to CFAR
Min/Max Noise	Percentage of Min/Max Noise removal while estimating background
Min/Max Deviation	Allowable Min/Max Standard Deviation of background to control clutter
Min/Max Difference	Ship central signal should be Min/Max DN more than background; to avoid unwanted detection.
Min/Max Ship	The range of Ship DN
Min/Max Water	The range of water DN
Water Occupancy	Minimum number of pixels from background are watery.
Ship Occupancy	Maximum number of pixels from Guard-band are of Ship DN
Reduction phase configurations	
Cloud Threshold	Minimum standard deviation of a block to be cloudy
Cloud Contour	n-layers of masking near cloud boundaries, to control thinner cloud spread
Cluster Area	Radius of cluster center
Cluster Cardinality	Maximum allowable ships under a Cluster Area

**Table 7:** Scene wise statistics.

Scene #	Operator Identified	System Identified	True +Ve	True −Ve	False +Ve	Precision (%)	Recall (%)
1	18	25	16	2	9	64	89
2	164	171	131	33	40	77	80
3	17	11	11	6	0	100	65
4	24	93	22	2	71	24	92
5	64	98	60	4	38	61	94
6	82	151	66	16	85	44	80
7	167	204	157	10	47	77	94
8	103	133	102	1	31	77	99
9	38	40	35	3	5	88	92
10	101	120	68	33	52	57	67
Total	778	1046	668	110	378	64	86
